# Prevalence and risk factors of Hepatitis C virus infection in Brazil, 2005 through 2009: a cross-sectional study

**DOI:** 10.1186/1471-2334-13-60

**Published:** 2013-02-01

**Authors:** Leila MMB Pereira, Celina MT Martelli, Regina C Moreira, Edgar Merchan-Hamman, Airton T Stein, Regina Maria A Cardoso, Gerusa M Figueiredo, Ulisses R Montarroyos, Cynthia Braga, Marília D Turchi, Gabriela Coral, Deborah Crespo, Maria Luiza C Lima, Luis Claudio A Alencar, Marcelo Costa, Alex A dos Santos, Ricardo AA Ximenes

**Affiliations:** 1 Universidade de Pernambuco, Faculdade de Ciências Médicas de Pernambuco, Hospital Universitário Oswaldo Cruz, Rua Arnóbio Marques, 310, Santo Amaro, CEP: 50100-130, Recife, PE, Brazil; 2 Instituto do Fígado de Pernambuco, Rua Aluísio Azevedo, 209, Santo Amaro, 50.100-130, Recife, PE, Brazil; 3 Universidade Federal de Goiás, Instituto de Patologia Tropical e Saúde Publica, Departamento de Saúde Coletiva, Rua Delenda Rezende de Mello, s/n, sala 405, Setor Universitário, CEP: 74605050, Goiânia, GO, Brazil; 4 Universidade Federal de Pernambuco, Departamento de Medicina Tropical, Av. Prof. Moraes Rego, s/n, Cidade Universitária, CEP: 50670-420, Recife, PE, Brazil; 5 Instituto Adolfo Lutz, Av. Dr. Arnaldo, nº 355, Cerqueira Cézar, CEP: 01246-902, Capital, SP, Brazil; 6 Universidade de Brasília, Faculdade de Ciências da Saúde, Departamento de Saúde Coletiva. DSC - Faculdade de Saúde, Campus Universitário Darcy Ribeiro – Asa Norte, CEP: 70910-900, Brasília, DF, Brazil; 7 Fundação Universidade Federal de Ciências da Saúde de Porto Alegre, Rua Sarmento Leite, 245, Bom Fim, CEP: 90050-170, Porto Alegre, RS, Brazil; 8 Departamento de Epidemiologia, Universidade de São Paulo, Faculdade de Saúde Pública, Avenida Dr. Arnaldo 715, Cerqueira Cesar, 01246-904, São Paulo, SP, Brazil; 9 Fundação Oswaldo Cruz, Centro de Pesquisas Aggeu Magalhães, Av Moraes Rego, s/n, Cidade Universitária, CEP: 50000-230, Recife, PE, Brazil; 10 Secretaria de Saúde Pública do Estado do Pará, Av. Conselheiro Furtado, 1597, CEP 66040-100, Belém, PA, Brazil; 11 Universidade Federal de Pernambuco, Departamento de Medicina Preventiva e Social, Av. Prof. Moraes Rego, s/n, Cidade Universitária, CEP: 50670-420, Recife, PE, Brazil; 12 Hospital de Base do DF, Area Especial, Asa Sul, CEP:70.335-900, Brasília, DF, Brazil; 13 Instituto Bioestatístico –IBCT, Rua Bernal do Couto, 1311, Umarizal, CEP: 67150-050, Belem, PA, Brazil

**Keywords:** Hepatitis C virus, Prevalence, Genotype, Cross sectional study, Risk factors

## Abstract

**Background:**

Hepatitis C chronic liver disease is a major cause of liver transplant in developed countries. This article reports the first nationwide population-based survey conducted to estimate the seroprevalence of HCV antibodies and associated risk factors in the urban population of Brazil.

**Methods:**

The cross sectional study was conducted in all Brazilian macro-regions from 2005 to 2009, as a stratified multistage cluster sample of 19,503 inhabitants aged between 10 and 69 years, representing individuals living in all 26 State capitals and the Federal District. Hepatitis C antibodies were detected by a third-generation enzyme immunoassay. Seropositive individuals were retested by Polymerase Chain Reaction and genotyped. Adjusted prevalence was estimated by macro-regions. Potential risk factors associated with HCV infection were assessed by calculating the crude and adjusted odds ratios, 95% confidence intervals (95% CI) and p values. Population attributable risk was estimated for multiple factors using a case–control approach.

**Results:**

The overall weighted prevalence of hepatitis C antibodies was 1.38% (95% CI: 1.12%–1.64%). Prevalence of infection increased in older groups but was similar for both sexes. The multivariate model showed the following to be predictors of HCV infection: age, injected drug use (OR = 6.65), sniffed drug use (OR = 2.59), hospitalization (OR = 1.90), groups socially deprived by the lack of sewage disposal (OR = 2.53), and injection with glass syringe (OR = 1.52, with a borderline p value). The genotypes 1 (subtypes 1a, 1b), 2b and 3a were identified. The estimated population attributable risk for the ensemble of risk factors was 40%. Approximately 1.3 million individuals would be expected to be anti-HCV-positive in the country.

**Conclusions:**

The large estimated absolute numbers of infected individuals reveals the burden of the disease in the near future, giving rise to costs for the health care system and society at large. The known risk factors explain less than 50% of the infected cases, limiting the prevention strategies. Our findings regarding risk behaviors associated with HCV infection showed that there is still room for improving strategies for reducing transmission among drug users and nosocomial infection, as well as a need for specific prevention and control strategies targeting individuals living in poverty.

## Background

Around the world, infection by the Hepatitis C virus (HCV) causes acute and chronic liver disease and may lead to cirrhosis, liver failure and/or hepatocellular carcinoma. HCV chronic liver disease is one of the main causes of liver transplantation in developed countries [1]. The global prevalence of HCV infection is estimated to be 2.2%, with 170 million individuals infected (2004) [2].

The sequential population-based studies conducted in the USA are among the few nationwide surveys to estimate the prevalence of HCV [3-6]. In the USA, a 1.6% nationwide prevalence of anti-HCV was found by the National Health and Nutrition Examination Survey Continuous (NHANES Continuous) between 1999 and 2002 [5]. These surveys showed a decline in the prevalence of hepatitis C from NHANES III (1988–1994) to NHANES Continuous (2003–2006), but this figure remained stable between the NHANES 1999–2002 and NHANES 2003–2006 [6]. The incidence of acute hepatitis C tended to decline over the twenty-five years of surveillance (1982–2006) in selected counties in the USA, with intravenous drug use (IDU) continuing to be the predominant risk factor [7]. In France, a population-based survey showed an overall prevalence of anti-HCV of 0.84% in 2004. IDU, nasal drug use, blood transfusion prior to 1992, having a tattoo, being older than 29 years, social deprivation, and being born on a continent with endemic levels higher than 2.5% were the risk factors associated with HCV infection [8].

In Latin America, estimates of HCV prevalence range from 1.0 to 2.3% using projections of HCV for modeling [9]. Data from the World Health Organization (WHO) placed Brazil among the countries with a prevalence of hepatitis C infection ranging from 2.5% to 10% [2]. Compulsory notification of acute and/or chronic hepatitis C was implemented by the Brazilian surveillance system in 1996. The detection rates for anti-HCV remained stable at approximately 10 cases per 100,000 individuals from 2004 to 2009, with the bulk of cases being reported in the southeast region [10]. In Brazil, the few population-based studies [11-13] and the vast majority of studies reporting risk factors associated with HCV infection were obtained from selected populations in restricted areas [14-18]. In a large sample of chronic HCV patients from different laboratories located in all Brazilian macro-regions, genotype 1 was found to predominate, followed by genotypes 3, 2, 4 and 5 [19].

A national seroepidemiological survey of Hepatitis A, B and C infection was launched by the Brazilian Ministry of Health in 2005 and completed in 2009. The epidemiology of hepatitis A and hepatitis B for the northeast and central-west regions and the Federal District has already been published [20,21]. The aim of the present study was to estimate the seroprevalence of HCV antibodies in the five macro-regions of Brazil and the Federal District. The association between potential risk factors and HCV seropositivity was assessed for the country as a whole and the population attributable risk for multiple factors was estimated using the case–control approach. This is the first population-based HCV antibody survey conducted in a representative sample of the Brazilian macro-regions and the Federal District.

## Methods

### Study design and sites

The national seroepidemiological survey of hepatitis A, B and C infection was designed as a representative sample of individuals aged 10 to 19 and 20 to 69 years in the 5 Brazilian macro-regions. Participants were residents of all 26 State capitals and the Federal District. The fieldwork was conducted from 2005 to 2009. The methodology has been described in a prior publication [22].

In brief, a stratified multistage cluster sampling method based on The Brazilian Institute of Geography and Statistics (IBGE) census data for the year 2000 was used to select the study population. In the first step, within each macro-region, the sample was stratified by state capital using a constant sampling fraction. In the second step the primary sampling units – census tracts – were drawn with probability proportional to population size and assuring the inclusion of all socioeconomic groups. The primary sampling units were subsampled successively in terms of blocks (with probability proportional to population size), households and eligible individuals. The final analysis weights incorporated the selection probabilities and design effect due to the cluster sampling to obtain representative estimates of the target population.

### Data collection and variables

All sites followed the same standard protocol. Data were collected by interview using a structured questionnaire during household visits. Blood samples were collected after the interviews. Both procedures were performed by trained health personnel.

The individual variables collected were social-demographic characteristics, blood route-related factors, such as dental treatment, surgery, history of blood transfusion, hospitalization, endoscopy, use of glass syringe not related with drug abuse, tattoos, or body piercing. Questions related to alcohol, drug consumption, lifelong sexual behavior and health-related jobs were restricted to persons aged 13 years or older. At the household level, data on income and education of the head of family and the characteristics of the household including home ownership, water supply, sewage and solid waste disposal were recorded [22].

### Serum tests and definitions

Blood samples were collected, transported and stored according to standard procedures. HCV antibodies were detected by a third-generation (Axsym, Abbott Laboratories, Abbott Park, Illinois) enzyme immunoassay in central public health laboratories. The results were considered positive when the optical density (OD) was ≥ 1.2 (20% higher than the cutoff). Samples with OD between 1.0 and 1.19 were considered indeterminate and were retested by anti-HCV; if the second testing yielded an indeterminate result, the sample was considered as negative. Seropositive individuals were submitted to a new blood sample collection to perform PCR and genotyping tests for clinical approach.

### HCV RNA detection and genotyping

HCV RNA was detected using AMPLICOR™ (Roche Version 2.0) and extracted from 200 μL serum using the extraction reagents included in the kit, in accordance with the manufacturer’s instructions.

A commercially available reverse hybridization-based line probe assay (HCV Genotype assay, LiPA™) was employed, using Amplicor’s PCR product generation. The genotypes were identified following the manufacturer’s instructions.

### Sample size

The sample size was based on the expected prevalence of seropositivity for anti-HCV according to data from blood donors by macro-region [23]. The variance was estimated as 20% of the prevalence value for all macro-regions and 30% for the Federal District [22]. The sample size achieved in this study matched the number of individuals estimated for each region and age group as previously reported.

As in the original protocol the analysis of risk factors for anti-HCV positivity was planned for the entire country, the number of seropositive individuals identified by the survey was large enough to detect differences in exposure corresponding to an odds ratio of 2, with a frequency of exposure among those not sick of 5% or more, a power of 80% and an alpha of 5%.

### Statistical analysis

Prevalence of anti-HCV with 95% confidence intervals (95% CI) was calculated for the country, by age group and by the five macro-regions and the Federal District. The estimated number of persons infected was based on the prevalence and on the census population official data of residents from the State Capitals by age groups for the year 2000 [24]. Prevalence was adjusted for the design effect and, when necessary, for regional variations in sample fractions.

The distributions of potential risk factors and their respective 95% CIs were calculated for the population aged 13 to 69 years old by macro-region. Possible associations between HCV infection and potential risk factors were assessed by calculating the Odds Ratio (OR), (95% CI) and p-values. ORs were adjusted for the design effect and age, and weighted for variations in sample fractions in the different age groups and regions. Variables that were associated with the outcome in this age-adjusted analysis (p < 0.10) were included in a multivariate regression model by means of the Generalized Linear and Latent Mixed Models (GLLAMM). The two-level analysis (individual and household) was performed using Stata 9.2 [25]. Population attributable risk—the fraction of disease in the total study population that is attributable to the exposure— was estimated for multiple factors using case–control data according to Bruzzi *et al*. [26]. Population attributable risk was also calculated for the ensemble of variables using an accepted procedure; it is worth noting that this estimate is not the equivalent of adding the population attributable risks for individual factors [26].

A ROC curve was plotted to predict viremia for the North macro-region, and to determine the cut-off for anti-HCV positivity using the Sample rate/cut-off rate (S/CO) ratios from the individuals tested for HCV-RNA.

### Ethical issues

Interviews and blood sample collection were conducted after written consent forms had been signed. For minors, the legal guardian’s consent was obtained. The project was approved by the National Research Ethics Committee (CONEP) of the Brazilian National Health Council and by the local Research Ethics Committees in each capital city.

Each positive result was communicated by the medical doctor in charge of the field research and the negative results were sent by mail. Individuals who had a positive result were sent to referral services in each State capital or the Federal District for further clinical evaluation.

## Results

Of 19,503 individuals aged 10 to 69 years old recruited, 2,288 were in the North region (Amazon area); 3,690 in the North-east; 3,702 in the Central-west; 1,988 in the Federal District; 3,661 in the South-east and 4,174 in the South. Of the total sample, 49.0% of the participants were aged 10–19 years old (n = 9,371) and 51.0% were 20–69 (n = 10,132). Females accounted for 54.6% of the total participants. The overall refusal rate was 2.8%, being below 1.5% for the North, Northeast and Central-west macro-regions and approximately 6% in the South and Southeast macro-regions.

In Brazil, the overall weighted prevalence of HCV antibodies was 1.38% (95% CI 1.12%–1.64%) in the state capitals of the five macro-regions and the Federal District taken as a whole (Table 1). Seropositivity varied from 0.68% in the Northeast to 2.10% in the North region. In all macro-regions studied, adults (aged 20–69 years) had a higher prevalence of HCV antibodies than the younger age-group. The highest prevalence in adults was in the North region (3.22%; 95% CI 2.03%–4.41%). Prevalence peaked in those aged ≥ 60 years.

**Table 1:** Prevalence of hepatitis C antibodies and estimated population ever-infected in a representative sample of individuals living in the state capitals of Brazil, 2005–2009

**Setting age-group**	**Participants**	Prevalence* % (95% CI)	**State capitals population**	**Estimated persons ever infected n (95% CI**)
Brazil				
10 – 19	9,371	0.75 (0.53 – 0.98)†	7,565,695	56,970 (39,947 – 74,068)
20 – 39	5,552	1.36 (1.02 – 1.71)†	13,828,140	188,478 (140,494 – 236,462)
40 – 59	3,719	1.55 (1.09 – 2.01)†	7,941,985	122,783 (86,250 – 159,396)
60 – 69	891	3.41 (2.03 – 4.79)†	1,916,570	65,432 (38,964 – 91,900)
North				
10 – 19	1,153	0.99 (0.36 –1.63)	860,922	8,601 (3,117 – 14,093)
20 – 69	1,135	3.22 (2.02 – 4.41)	2,093,269	67,403 (42,472 – 92,334)
Northeast				
10 – 19	1,840	0.38 (0.10 – 0.66)	2,054,539	7,848 (2,096 – 13,601)
20 – 69	1,850	0.97 (0.48 – 1.47)	5,358,923	52,249 (25,616 – 78,883)
Midwest				
10 – 19	1,803	0.99 (0.53 – 1.47)	866,536	8,657 (4,601 – 12,712)
20 – 69	1,899	1.64 (1.14 – 2.13)	1,326,575	21,703 (15,110 – 28,296)
Federal District				
10 – 19	980	0.61 (0.13 – 1.09)	387,831	2,374 (524 – 4,227)
20 – 69	1,008	1.09 (0.50 – 1.68)	1,202,183	13,116 (6,011 – 20,245)
Southeast				
10 – 19	1,794	0.90 (0.49 – 1.31)	3,295,110	29,755 (16,212 – 43,298)
20 – 69	1,867	1.63 (1.07 – 2.18)	11,661,984	189,857 (124,783 – 254,814)
South				
10 – 19	1,801	0.51 (0.12 – 0.89)	586,270	2,978 (715 – 5,241)
20 – 69	2,373	1.70 (1.08 – 2.32)	2,043,761	34,826 (22,175 – 47,456)

* Prevalence adjusted for random effect.

† Prevalence adjusted for random effect and weighted by regions.

The estimated number of persons ever infected in the Brazilian State capitals and Federal District was 430,658 (95% CI 349,714–511,289), based on the HCV prevalence and the size of the population. The largest infected population was in the Southeast region (190,254; 95% CI 136,110–244,548).

**Figure 1:**
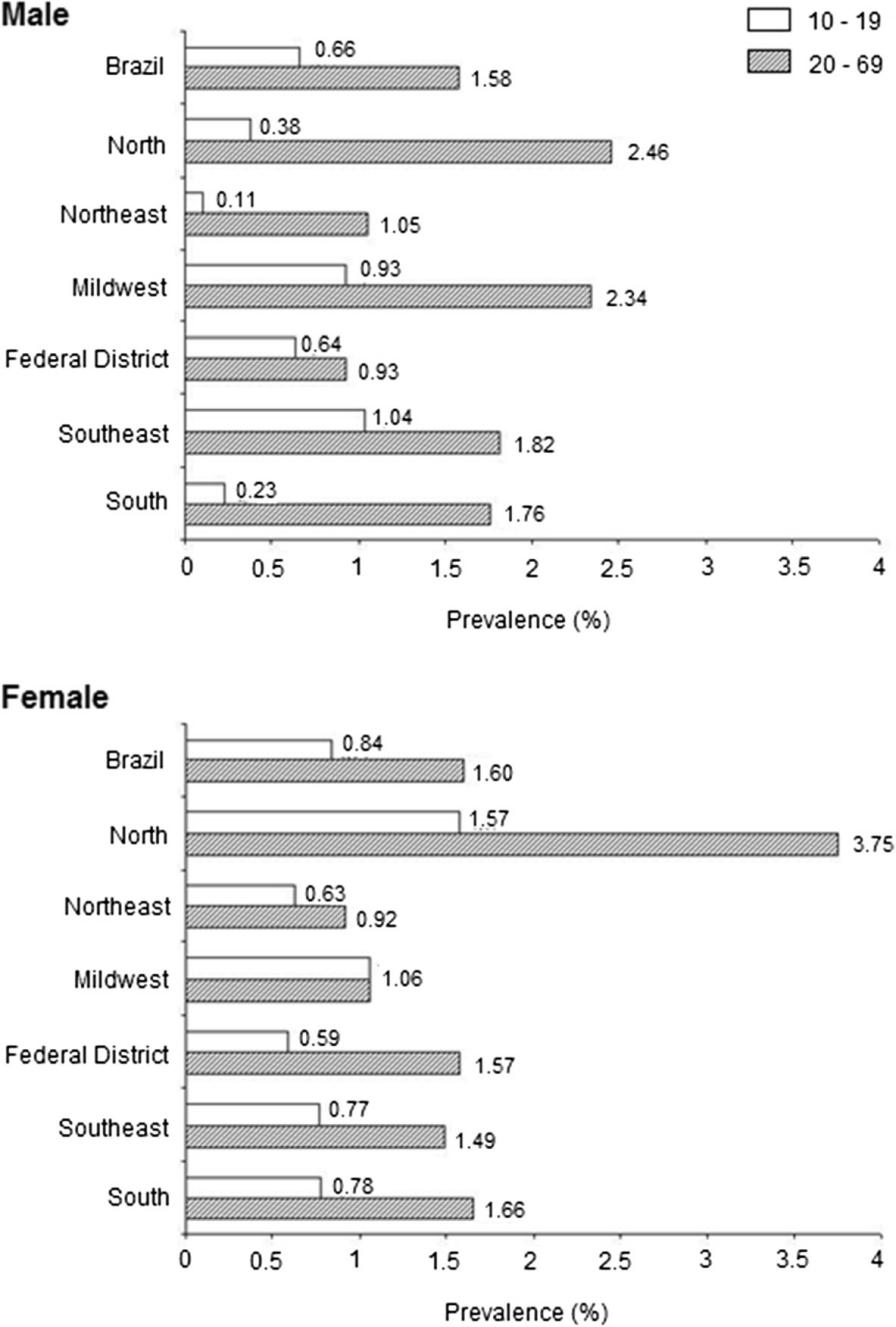
Prevalence of hepatitis C infection in a representative sample of State capitals of the five Brazilian regions by sex and age group.

For the overall data set, anti-HCV positivity was similar between genders according to the two age strata (Figure 1). When stratified by macro-region, anti-HCV prevalence was similar between men and women in most of the studied settings, except in the Central-West macro-region, where men had a statistically significant higher prevalence of HCV infection than women (chi-square = 4.9, p < 0.03) (Figure 1).

Of 9,671 households investigated, ten contained two individuals testing positive for anti-HCV and one household contained three. Thirty participants were simultaneously seropositive for anti-HCV and anti-HBc antibodies.

Of 236 anti-HCV positive individuals, 120 (50.8%) had their blood collected for HCV-RNA testing in all the macro-regions and Federal District, with the exception of the North macro-region, owing to operational constraints. Of those retested, 36.7% (95% IC 28.4%-45.6%) were HCV-RNA positive. The ROC curve suggested that the cut-off point of 0.4, which yielded the higher sensitivity and specificity, was OD = 4.0. Using this parameter, 32.1% individuals would also be considered infected, that is, HCV-RNA positive, in the North region. The comparison of the participants who were retested (n = 120) versus those not retested (n = 116) in relation to variables associated with HCV infection that remained in the multivariate model showed that both groups were similar in terms of age group, use of injected and sniffed drugs, use of glass syringes and hospitalization. The main difference was in socio-economic conditions (chi-square = 8.8, p = 0.012) suggesting that those better-off were more likely to be retested. Assuming the frequency of HCV-RNA-positive individuals among the estimated persons ever infected in the present study, it would be expected that there were approximately 158,000 (122,000 to 196,000) patients infected with HCV.

The genotypes and subgenotypes detected were 1 (n = 3); 1a (n = 9); 1b (n = 16); 2b (n = 2); 3a (n = 14).

Tables 2, 3, 4 show the age-adjusted odds ratio for the association between blood-related variables, sexual behavior and drug use and HCV infection for all the State capitals taken together. There was a positive association between anti-HCV antibodies and ever having received a blood transfusion (OR = 1.83, 95% CI 1.01–3.30), having been hospitalized in the previous 12 months (OR = 1.87, 95% CI 1.04–3.38), or having had a tattoo (OR = 1.63, 95% CI 0.95–2.80), the latter presenting a borderline p value (Table 2). Having a current partner with hepatitis was positively associated with HCV antibodies and negatively associated with partner history of STD (Table 3). Ever having used injected drugs was strongly associated with HCV antibodies (OR = 12.79, 95% CI 5.19–31.50). A statistically significant association was also found with ever having used sniffed drugs (OR = 3.50, 95% CI 1.87–6.54) and previous use of a glass syringe (OR = 1.67, 95% CI 1.09–2.56) (Table 4). As for socioeconomic variables, sewage disposal (lack of sewage system versus public system) was associated with HCV antibodies (OR = 1.90, 95% CI 1.06–3.40).

**Table 2:** Age-adjusted odds ratio of hepatitis C infection for blood route-related factors in the urban Brazilian population

**Risk factors**	**Participants**	**HCV infection n (%)**	**OR**_**adjusted **_(CI)*	**p-value**
Dental treatment				
Never	2,034	22 (1.1)	1.0	-
Past 12 months	8,655	99 (1.1)	0.77 (0.41 – 1.47)	0.436
Ever	8,772	114 (1.3)	0.91 (0.47 – 1.77)	0.795
Surgery				
Never	11,481	108 (0.9)	1.0	-
Past 12 months	1,689	31 (1.8)	1.38 (0.72 – 2.64)	0.327
Ever	6,270	96 (1.5)	0.93 (0.60 – 1.42)	0.730
Blood transfusion				
Never	18,122	196 (1.1)	1.0	-
Past 12 months	256	10 (3.9)	1.45 (0.65 – 3.21)	0.365
Ever	843	24 (2.9)	1.83 (1.01 – 3.30)	0.043
Hospitalization				
Never	13,095	143 (1.1)	1.0	-
Past 12 months	1,308	25 (1.9)	1.87 (1.04 – 3.38)	0.037
Ever	5,032	67 (1.3)	1.46 (0.97 – 2.19)	0.065
Endoscopy				
Never	16,695	190 (1.1)	1.0	-
Past 12 months	904	13 (1.4)	1.14 (0.53 – 2.47)	0.729
Ever	1,785	29 (1.6)	1.17 (0.64 – 2.15)	0.615
Tatoo				
No	18,034	202 (1.1)	1.0	-
Yes	1,459	33 (2.3)	1.63 (0.95 – 2.80)	0.076
Body piercing				
No	17,859	215 (1.2)	1.0	-
Yes	1,634	20 (1.2)	0.97 (0.51 – 1.87)	0.935
Health-care related job				
No	15,424	195 (1.3)	1.0	-
Yes	1,206	20 (1.7)	0.91 (0.46 – 1.80)	0.783

* Adjusted for random effect and age weighted by age groups and regions.

**Table 3:** Age-adjusted odds ratio of hepatitis C infection for sexual behavioral-related factors in the urban Brazilian population

**Risk factors**	**Participants**	**HCV infection n (%)**	**OR**_**adjusted **_(95% CI)*	**p-value**
Initiated sexual life				
No	4,137	36 (0.9)	1.0	-
Yes	12,468	179 (1.4)	0.97 (0.56 – 1.68)	0.912
Condom use				
Always	3,831	37 (1.0)	1.0	-
Sometimes	1,944	29 (1.5)	1.71 (0.85 – 3.43)	0.132
Never	6,077	103 (1.7)	1.04 (0.56 – 1.96)	0.888
Current partner had hepatitis				
No	10,263	140 (1.4)	1.0	-
Yes	445	13 (2.9)	2.16 (1.00 – 4.65)	0.050
Bisexual partner				
No	11,417	158 (1.4)	1.0	-
Yes	276	4 (1.4)	0.83 (0.29 – 2.42)	0.737
Unknown	775	17 (2.2)	1.41 (0.69 – 2.87)	0.343
Sexual partners with STD				
No	11,086	164 (1.5)	1.0	-
Yes	819	10 (1.2)	0.43 (0.20 – 0.93)	0.031
Previous STD				
No	11,079	153 (1.4)	1.0	-
Yes	1,301	25 (1.9)	0.96 (0.50 – 1.83)	0.901
Another sexual partner besides current				
No	4,703	64 (1.4)	1.0	-
Yes	6,978	101 (1.4)	1.10 (0.73 – 1.65)	0.657

* Adjusted for random effect and age weighted by age groups and regions.

**Table 4:** Age-adjusted odds ratio of hepatitis C infection for drug use-related factors in the urban Brazilian population

**Risk factors**	**Participants**	**HCV infection n (%)**	**OR**_**adjusted **_(95% CI)*	**p-value**
Ever use of smoked drugs				
No	14,726	177 (1.2)	1.0	-
Yes	1,839	38 (2.1)	1.47 (0.88 – 2.46)	0.141
Ever use of inhalated drugs				
No	16,293	206 (1.3)	1.0	-
Yes	265	09 (3.4)	2.68 (0.85 – 8.45)	0.093
Ever use of sniffed drugs				
No	15,827	189 (1.2)	1.0	-
Yes	749	25 (3.3)	3.50 (1.87 – 6.54)	0.000
Ever use of injected drugs				
No	16,463	204 (1.2)	1.0	-
Yes	70	11 (15.7)	12.79 (5.19 – 31.5)	0.000
Injections with glass syringe				
No	13,450	148 (1.1)	1.0	-
Yes	2,931	63 (2.2)	1.67 (1.09 – 2.56)	0.019
Alcohol consumption				
None	10,237	129 (1.3)	1.0	-
Light consumption	5,376	67 (1.2)	0.75 (0.50 – 1.12)	0.155
Heavy consumption	978	16 (1.6)	0.84 (0.41 – 1.69)	0.619
Dependent	41	03 (7.3)	2.88 (0.79 – 10.49)	0.108

* Adjusted for random effect and age weighted by age groups and regions.

The multivariate analysis shows that a history of injected drug use (OR = 6.65; 95% CI 2.47–17.91) and sniffed drug use (OR = 2.59; 95% CI 1.34–5.81) were strongly associated to HCV infection. Other risk factors were the use of a glass syringe (OR = 1.52; 95% CI 0.97–2.36) and ever-hospitalization, both with a borderline p value. A proxy measure of socioeconomic conditions (sewage disposal) was also associated with testing positive for anti-HCV. Age, introduced in the model as a continuous variable, showed that there was an increase in the likelihood of becoming infected of 0.02% for each year of life (OR = 1.02; 95% CI 1.01–1.04 (Table 5). The proportion of cases attributable to each variable in the final multivariate model ranged from 4.2% to 10.2%, and the proportion attributable to all factors together was 39.9%.

**Table 5:** Multivariate analysis of hepatitis C infection for potential risk factors and estimated population attributable risk in the urban Brazilian population

**Factors associated**	**OR**_**adjusted **_(95% CI)*	**p-value**	**Population Arributable risk %**
Age	1.02 (1.01 – 1.04)	0.001	-
Ever use of injected drugs			
No	1.0	-	
Yes	6.65 (2.47 – 17.91)	< 0.0001	4.3
Ever use of sniffed drugs			
No	1.0	-	
Yes	2.59 (1.34 – 5.01)	0.005	7.2
Injection with glass syringe			
No	1.0	-	
Yes	1.52 (0.97 – 2.36)	0.065	10.2
Hospitalization			
Never	1.0	-	
Past 12 months	1.90 (1.03 – 3.51)	0.040	5.0
Ever	1.44 (0.94 – 2.21)	0.094	8.7
Sewage disposal			
Public system	1.0	-	
Septic tank	1.39 (0.91 – 2.12)	0.125	8.8
Other destination	2.53 (1.38 – 4.65)	0.003	3.9
			39.9**

* Adjusted for random effect and age weighted by age groups and regions.

** Summary population attributable risk.

## Discussion

This first Brazilian nationwide hepatitis survey showed 1.38% HCV positivity in a representative sample of 19,503 adolescents and adults in all macro-regions. Approximately 1.3 million individuals would be expected to be anti-HCV-positive in the country under the assumption of similar anti-HCV antibody prevalence for the Brazilian population as a whole (population of 169.8 million, as of 2000). The current population-based survey detected a lower prevalence of anti-HCV antibody than that previously reported by the WHO for the country [2], shifting from intermediate to low prevalence.

The few nationwide population-based studies have reported prevalence of anti-HCV antibodies of 1.3% in the USA [6] and 1.0% in France [27], which are similar to that found in this study. An increase in anti-HCV prevalence with age was found in the large census surveys conducted in the USA. Peak HCV prevalence was found among adults aged 30-49 years in the first census survey (1988–1994), shifting to 40–49 years in the later surveys (1999–2002; 2003–2006; 2007–2008) [6]. Although our results also showed increased HCV exposure with age, the peak prevalence was observed in the older age group. Increased HCV infection among the adult population has also been reported in selected regions of Brazil [11,13] and among first-time blood donors [28].

The highest prevalence of the HCV marker (2.1%) was in the North macro-region of Brazil, which is below the 2.5% prevalence threshold for low endemicity according to WHO criteria [2]. However, most of the estimated infected individuals resided in the Southeast region (~220,000), since approximately 14 million individuals aged 10 to 69 years live in these highly urbanized State capitals that include the cities of Sao Paulo and Rio de Janeiro. The burden of infection in absolute terms thus has an uneven geographical distribution, concentrated in the South and Southeast macro-regions. According to the official surveillance system, the residents of the southeast and south had higher HCV detection rates (above the national figures) than the residents in the north, northeast and central-west from 2003 through 2009 (unpublished data). One likely explanation for the higher detection rates in the south and southeast may be better access to and organization of the health system of these regions [29]. The surveillance data for HCV may also underestimate the prevalence of HCV infection in the country as a whole, in view of the prevalence found by this population-based survey. Under-reporting of HCV infection by other national surveillance systems has also been discussed for Europe [30,31] and for the USA [32], pointing to the need for updated population-based studies for public health purposes.

Interestingly, those participating in this national survey showed similarities in terms of the risk profile for HCV acquisition, with slightly higher frequencies for blood transfusion (7.8%), tattoos (12.2%) and sniffed drugs (5.9%) in the south macro-region (data not shown). In this household survey, injected and sniffed drug use was strongly associated with HCV antibodies in multivariate analysis. These findings accord with the literature regarding injected drug use as the main source of transmission of HCV infection in developed countries and with the reports of sniffed drugs as another important mode of HCV acquisition in developing countries, as previously reported in selected Brazilian populations [33]. In the present survey, a history of previous hospitalization and use of injections with glass syringe not related to drug use were also associated with HCV seropositivity, both of which have been widely reported as epidemiological risk factors for HCV infection worldwide [1,2]. In our survey, a socioeconomic marker of extreme poverty (no sewage disposal) remained associated with anti-HCV prevalence (OR = 2.53), after adjusting for confounding factors. Although there is no biological plausibility for social deprivation and increased risk of HCV infection, the former may be a surrogate marker for being at risk for HCV transmission. In a countrywide survey in France, social factors remained associated with HCV infection after adjusting for known risk factors as in our study. Nevertheless, having ever used drugs, having tattoos, and having being incarcerated were correlated with social deprivation [8]. The USA HCV prevalence survey also reported a correlation of infection by drug use with low family income, high number of sex partners, and non-injected drug use [5]. Furthermore, studies using cross-sectional designs such as our survey cannot sort out the temporal relationships between the potential risk factors and the outcomes.

The estimated population attributable risk indicates that the risk factors identified explain approximately 40% of all cases of infection. The higher population attributable risk percent found for “ever use of sniffed drugs” (7.2%) than for “ever use of injected drugs” (4.3%) was probably due to the lower frequency of IDUs than of sniffed drug users in the studied population. Although we cannot evaluate to what extent there was an underreporting of injection drug use because of stigma and discrimination in our study, the frequencies we found (data not shown) seem consonant with the national survey conducted by the Brazilian Center of Information on Psychotropic drugs (CEBRID) and a study among hospitalized patients [34,35]. In relation to IDU, as the population attributable risk is related to the general population (injecting and non-injecting drug users), the attributable risk among the exposed (injecting drug users) will be greater and therefore the proportion of infection in that group that could be prevented by eliminating the exposure would be larger among IDUs.

One limitation of this survey is that it included a sampling of the capital cities of the five Brazilian macro-regions and the Federal District and was not designed as part of a census survey [22] as was NHANES [3]. Therefore, the low prevalence of anti-HCV detected in all macro-regions may not reflect the levels of infection outside the study settings, such as in rural areas, and specific pockets. Since this investigation was planned as a population study, specific high-risk groups for HCV acquisition, such as institutionalized or incarcerated individuals, were excluded. Therefore, the prevalence reported here should be considered a conservative estimate of anti-HCV prevalence.

It should be noted that, in view of the low frequency of HCV antibodies for the country as a whole, the risk factor analysis was performed for the entire data set and not by macro-region. This had been anticipated by the study protocol [22].

Another methodological issue regarding the interpretation of our findings is that a sole marker of HCV infection may lead to misclassification, owing to false positive results. However, it would be a non-differential misclassification, underestimating the associations reported. For this reason, the strength of association may also be conservative [36]. We acknowledge that the approximately 40% of HCV-RNA positivity among anti-HCV positive individuals was lower than expected. Potential explanations for this are poor specificity of the anti-HCV antibody assay, poor sensitivity of the HCV-RNA assay, or a true biologically higher HCV-RNA spontaneous clearance rate in the Brazilian setting. In our study, high sensitivity (poor specificity) of anti-HCV was identified because of the cutoff point recommended by the manufacturer; this may have increased false positive rates. In this regard, the ROC curve showed that a cutoff point of OD =4.0 yielded higher specificity on the detection of viremia. The assay used (AMPLICOR™ Roche Version 2.0) for HCV-RNA testing detects 60 International Units per ml. A concern on the detection of hepatitis C virus RNA in a large population survey is the specimen handling and storage. Nevertheless, we have followed the laboratory procedures and storage recommendations and all tests were performed in a reference laboratory where quality control was assured. In addition, data analysis showed that the frequency of viremia found was consistent across macro-regions. In general, about 25% of infected HCV individuals have spontaneous viral clearance. A systematic review of longitudinal studies on spontaneous viral clearance showed that clearance was more frequent in females and in acute clinical hepatitis C infection [37]. Furthermore, a genome-wide association study showed that individuals with favorable *IL28B* genotypes are more likely to clear HCV infection than are those with unfavorable alleles, which could have implications in viral clearance ability among different ethnic groups [38]. In the South of Brazil, IL28B polymorphism was associated with spontaneous clearance of hepatitis C infection among co-infected HCV and HIV/AIDS patients [39].

Although this survey covered a large geographical area and enrolled a large population sample in all State Capitals and Federal District, there was low refusal rate in all macro-regions, which was slightly higher in the wealthier regions: South and Southeast. This indicates the feasibility of nationwide-studies.

In this survey, subtypes 1a, 1b, 2b and 3a were detected in the general Brazilian population within the macro-regions. Overall, genotype 1 was predominant, as reported in the literature, which suggests that genotype 1a and 1b are the most frequent HCV genotypes worldwide, accounting for 60% of the infections [2]. The geographical distribution of hepatitis C virus genotypes in Brazil among a large sample of chronic hepatitis C patients from all regions has also shown a predominance of genotype 1 followed by genotype 3 [19], in accordance with our findings. Among injecting drug-users, the circulating subtypes were 1 and 3 in the studies conducted between 1994–1997 and 1999–2001 in Rio de Janeiro [40]. Our study was not designed to identify the diversity of HCV genotypes among the regions. We are aware that not all anti-HCV positive individuals were tested for HCV-RNA, which may limit the extrapolation of these results.

## Conclusions

The present survey measured the hepatitis C infection prevalence among the general population for the country and macro-regions, and classifies Brazil as a low-endemicity area. Nevertheless, the large estimated absolute numbers of infected individuals indicate the burden of the disease in the near future. This implies costs for the health-care system and society as a whole, as pointed out in other studies [41,42]. This issue needs to be addressed by studies that evaluate the economic impact of HCV infection. The known risk factors explain less than 50% of the infected cases, limiting the prevention strategies. Nevertheless, our findings regarding risk behaviors associated with HCV infection showed that there is still room for improving strategies for reducing transmission among drug users. Another recommendation would be to study the extent to which nosocomial transmission is occurring in our setting. Finally, there is a need for targeting individuals living in poverty, in view of the evidence found of an association between infection and social deprivation.

## Abbreviations

HCV: Hepatitis C Virus; NHANES: National Health and Nutrition Examination Survey; IDU: Intravenous drug use; WHO: World Health Organization; IBGE: The Brazilian Institute of Geography and Statistics; OD: Optical density; RNA: Ribonucleic acid; PCR: Polymerase Chain Reaction; OR: Odds ratio; 95%CI: 95% Confidence Interval; GLLAMM: Generalized Linear and Latent Mixed Models.

## Competing interests

The authors declare that have no competing interests.

## Authors’ contributions

RAAX, LMMBP, CMTM, RAC, GMF designed the study, data analysis and drafted the manuscript. RCM and GMF coordinated the laboratory procedures and quality control. RAAX and URM were responsible for data management and statistical analysis. CB, MLCL, HH, ATS, AAS coordinated and supervised the fieldwork in macro-regions, participated in the data analysis and reviewed the manuscript. GC, MDT, DC and LCAA were in charge of the medical care for HCV positive participants, for case investigation/management and guidance. They also reviewed the manuscript. All authors read and approved the final version of this manuscript.

## Pre-publication history

The pre-publication history for this paper can be accessed here:

http://www.biomedcentral.com/1471-2334/13/60/prepub
